# Genetic background effects on meiotic recombination

**DOI:** 10.17912/micropub.biology.001247

**Published:** 2024-07-03

**Authors:** Ayah Lababede, Bowen Man, Nicole Crown

**Affiliations:** 1 Department of Biology, Case Western Reserve University, Cleveland, Ohio, United States

## Abstract

Genetic background is a strong driver of phenotype, and when analyzing a mutant phenotype, it is critical that a genetically comparable wildtype stock is used as a control. Many of the *Drosophila melanogaster* meiotic mutants were isolated in EMS screens or created by P-element mutagenesis and do not have a congenic or isogenic wildtype control. We show here that the *y ^1^* stock, commonly used for transgenesis in *D. melanogaster* , shows significantly higher meiotic crossover rates, and there is likely a modifier of meiosis or germline development in the genetic background of this popular stock.

**Figure 1. Analysis of meiotic recombination in the Drosophila yellow 1 stock.**
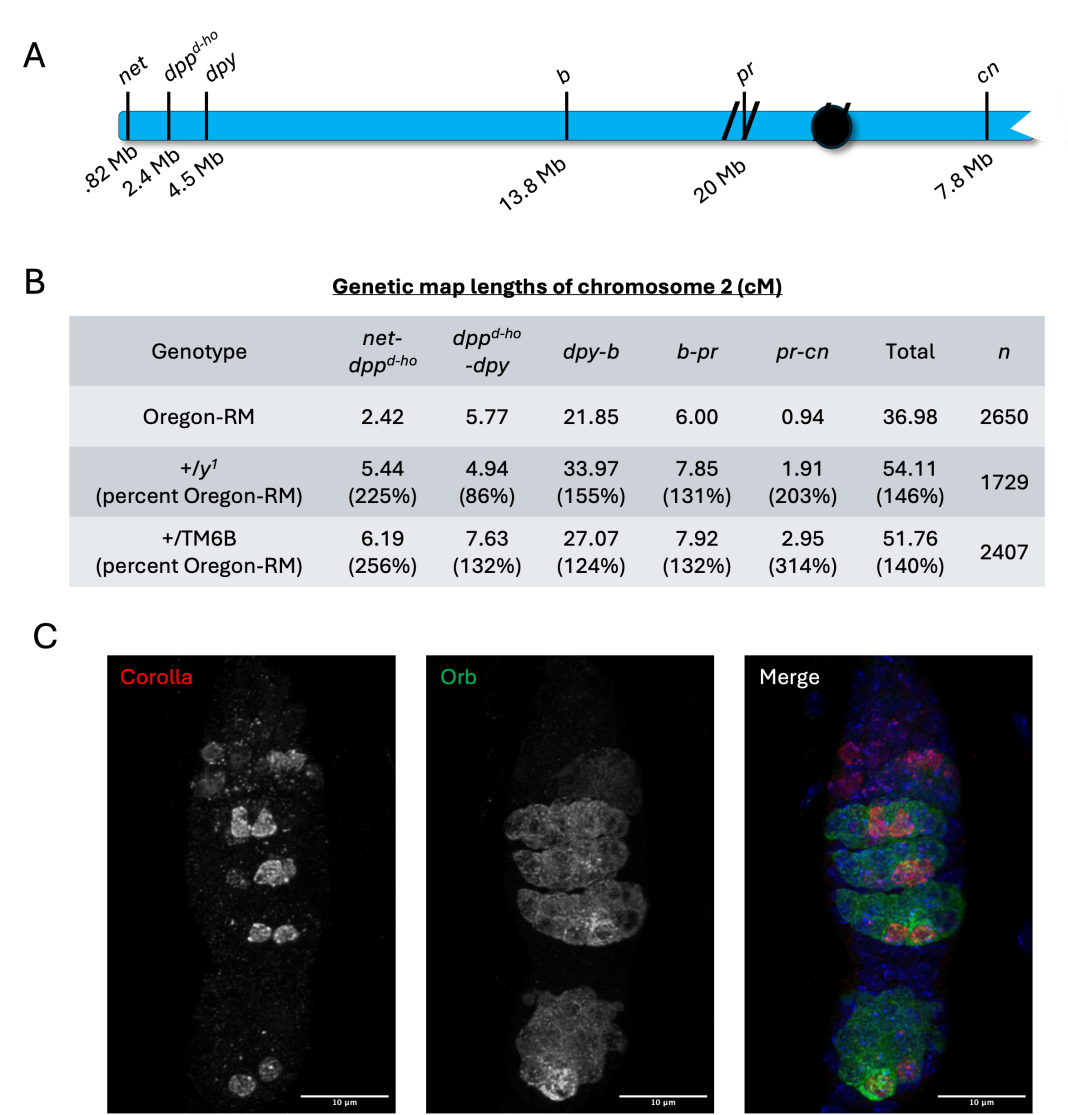
A) Map of chromosome 2 showing location of the recessive phenotypic markers used to determine crossover frequencies. Physical location along the chromosome is shown in Mb below. Hashes represent unmapped pericentromeric heterochromatin. B) Crossover frequencies for each genetic interval in Oregon-RM, +/ *y ^1^* , +/TM6B. Numbers in parentheses show the percent increase compared to Oregon-RM. All intervals show a statistically significant increase in frequency except the *dpp ^d-ho^ -dpy* interval in +/ *y ^1^* (Fisher’s exact test). C) Corolla and Orb staining the a +/ *y ^1^* germarium. Of the 15 germaria analyzed, 11 had two oocytes in region 3 at the bottom of the germarium as indicated by the presence of two corolla positive nuclei. Images are maximum projections.

## Description

Genetic background is a strong driver of phenotype. Because of this, the genetic background of a mutation needs to be controlled for by using an isogenic stock or creating a congenic stock by out-crossing a mutant into a shared wildtype genetic background. Unfortunately, across different model organism communities, many of the controls do not truly share a genetic background with the mutant being analyzed. For example, if a mutant was generated by injecting a genome engineering construct into a *w ^1118^* background, then *w ^1118^* will often be used as a control. However, most often, labs will often use their lab copy of *w ^1118^* and not the specific stock that was injected into, nor will labs use full siblings of the newly made mutant stock. However, sequencing efforts have shown that stocks kept isolated in individual labs accumulate unique sets of mutations, such that one lab’s copy of *w ^1118^* is not the same genetic background as another lab’s (Miller et al 2016) . Additionally, there are many mutants that are decades old for which there simply is not information on the genetic background.

In Drosophila, many of mutants that affect meiotic crossover formation were isolated from EMS mutagenesis screens or P-element mutagenesis many decades ago. Chromosome rearrangements such as inversions, translocations, and large deficiencies are also used to study meiosis, and these types of stocks were often the result of irradiation or genome engineering (Kaufman 2017) . Combined, the set of meiotic mutants in Drosophila come from a wide range of genetic backgrounds, yet in most experiments, the control crossover frequencies either come from an Oregon-RM or *w ^1118^* stock, or the standard genetic map is used. Importantly, with rare exception (for example Li et al 2023), the controls used in these studies are not usually congenic with the genetic background of the meiotic mutant stocks or the chromosome rearrangement stocks. This does not negate the use of these stocks as controls, as mutations that completely prevent crossovers from forming are unlikely to be due to genetic background; however, it may be crucial to use congenic controls when analyzing more subtle phenotypes such as changes in the crossover distribution.

We set out to test whether the meiotic crossover frequency and distribution of the *y ^1^* stock is similar to other wildtype stocks such as Oregon-RM. *y ^1^* is a common genetic background used for Drosophila transgenesis as transgenes are often marked with a wildtype copy of the *yellow* gene and injected into a mutant *yellow* background. To mimic a standard experiment where crossover frequencies are being analyzed, we generated females that were heterozygous for *y ^1^* and heterozygous for a second chromosome that has multiple recessive phenotypic markers (+/ *y ^1^* ; +/ *net ^1^ dpp ^d-ho^ dpy ^ov1^ b ^1^ pr ^1^ cn ^1^* ). These heterozygous females were crossed to homozygous *net-cn* males, and the offspring were scored for each recessive marker. Crossovers occurred between markers that switched from wildtype to mutant phenotypes.

To our surprise, we found that the overall map length in the *net-cn* region of chromosome 2 was 54.11 cM compared to 36.98 cM in Oregon-RM (a 46% increase in crossover frequency, p = 0.0001, Fisher’s exact test, Figure 1 ). Furthermore, we noticed the largest increases occurred in the centromere spanning interval ( *pr-cn* ) and the subtelomeric interval ( *net-ho* ), with only moderate increases in the medial intervals.

The pattern of increase and the overall map length increase seen in the *y ^1^* background is identical to the type of increase that occurs during the interchromosomal effect (Lucchesi and Suzuki 1968) . The interchromosomal effect occurs in some Drosophila species when the presence of a heterozygous inversion suppresses crossovers on the inversion pair but increases the crossover frequency in the remainder of the genome due to activation of the pachytene checkpoint (Joyce and McKim 2010) . The increase in crossover frequency is not uniform, with the majority of increases occurring in the centromere spanning and subtelomeric intervals (Lucchesi and Suzuki 1968) . To confirm that the change in crossover frequency and distribution was similar to the interchromosomal effect in our lab stocks, we generated females that were heterozygous for the same *net-cn* second chromosome and heterozygous for TM6B, a widely used multiply inverted balancer chromosome. Indeed, we found that the crossover frequency and distribution on chromosome 2 was not statistically different between *y ^1^* and TM6B (p = 0.15, Fisher’s exact test) raising the possibility that there is an interchromosomal effect in the *y ^1^* stock.

To further test whether there is an interchromosomal effect in *y ^1^* , we analyzed the two-oocyte frequency. In the Drosophila germarium, stem cells differentiate into cystoblasts. These cystoblasts undergo four rounds of mitosis with incomplete cytokinesis, resulting in 16-cell cysts. Within each cyst, up to four cells initiate meiosis, but by the end of the germarium, only one cell is designated as the oocyte and the other cells exit meiosis (Hughes et al 2018) . When the pachytene checkpoint is activated by the presence of a heterozygous inversion, there is also a delay in oocyte specification and there are two oocytes at the end of the germarium instead of one (Joyce and McKim 2010) . This serves as a cytological readout for activation of the pachytene checkpoint and indirectly of the interchromosomal effect. We measured the two-oocyte phenotype by staining for Corolla (a member of the synaptonemal complex that marks meiotic nuclei, Collins et al 2014) and Orb (a cytoplasmic RNA binding protein that marks the final oocyte, Lantz et al 1994). We found that in *y ^1^* heterozygotes, 11 out of 15 germaria had two oocytes, suggesting that the pachytene checkpoint has been activated.

Our results show that the overall crossover frequency on chromosome 2 in *y ^1^* heterozygotes is 46% higher than Oregon-RM with some intervals having much larger increases than others, they are not distributed with the same pattern, and there is a delay in oocyte specification. While our data are suggestive of the interchromosomal effect occurring in *y ^1^ ,* we have not identified the causal modifier in this stock. The interchromosomal effect can also be caused by translocations and presumably other types of structural variants (Joyce and McKim 2010, Lucchesi and Suzuki 1968) . Additionally, the pachytene checkpoint can be activated by developmental defects that are independent of meiotic chromosome dynamics (Pek et al 2012) . Lastly, mutations in C(3)G cause changes in crossover frequency and distribution strikingly similar to the interchromosomal effect (Billmyre and Cahoon et al 2019) . Despite not knowing what the modifier is in this genetic background, we have clearly shown the *y ^1^* stock does not undergo a wildtype meiosis compared to other domesticated wildtype lab stocks. Our results are in line with previous data showing that genetic background influences when the synaptonemal complex is disassembled (Wesley et al 2020) . Together, these results clearly demonstrate that it is essential to control for genetic background when analyzing meiosis or any biological process in Drosophila, particularly when analyzing subtle phenotypes as variation in baseline crossover rates might obscure or enhance differences in crossover frequencies.

## Methods

Drosophila husbandry

All stocks were maintained at 25 degrees Celsius on standard cornmeal media. The *net ^1^ dpp ^d-ho^ dpy ^ov1^ b ^1^ pr ^1^ cn ^1^* , Oregon-RM, *y ^1^* , and TM6B stocks have been maintained in the Crown lab since 2018 and are available upon request.

Crossover scoring

We crossed females that were Oregon-RM to *net ^1^ dpp ^d-ho^ dpy ^ov1^ b ^1^ pr ^1^ cn ^1^* males and recovered +/Oregon-RM; +/ *net ^1^ dpp ^d-ho^ dpy ^ov1^ b ^1^ pr ^1^ cn ^1^* females. These females were crossed to *net ^1^ dpp ^d-ho^ dpy ^ov1^ b ^1^ pr ^1^ cn ^1^* males and the offspring were scored for crossovers. This same process was repeated for the *y ^1^* stock. To analyze the interchromosomal effect, we crossed TM3 Sb/TM6B Hu Tb females to *net ^1^ dpp ^d-ho^ dpy ^ov1^ b ^1^ pr ^1^ cn ^1^* males and recovered +/ *net ^1^ dpp ^d-ho^ dpy ^ov1^ b ^1^ pr ^1^ cn ^1^ ;* +/TM6B Hu Tb females. These females were crossed to *net ^1^ dpp ^d-ho^ dpy ^ov1^ b ^1^ pr ^1^ cn ^1^* males and the offspring were scored for crossovers. Crosses were set up in vials with 5 females and 2 males to avoid any batch effects.

Immunofluorescence

2-3 day old mated females were put on yeast paste overnight. Ovaries were dissected in PBS and fixed for 20 minutes in 1000 ml of solution containing 2% paraformaldehyde (Ted Pella cat. no. 18505), 0.5% Nonidet P-40 (Sigma cat. no. I8896), 200 ml PBS and 600 ml heptane. Ovaries were then washed three times for ten minutes each in PBS with 0.1% Tween-20 (PBST), blocked for one hour at room temperature in PBS with 1% BSA (MP Biomedicals cat. no. 152401) and incubated with primary antibody diluted in PBST overnight at 4 degrees. Ovaries were then washed three times in PBST and incubated in secondary antibody diluted in PBST for 4 hours at room temperature. DAPI was added for the last 10 minutes at a concentration of 1 ml/ml. Ovaries were washed again three times for 15 minutes each in PBST. All wash steps and antibody incubations were done while nutating. Ovaries were mounted in ProLong Glass (Invitrogen cat. no. P36980) and allowed to cure for the manufacturer’s suggested time. Antibodies were anti-Corolla (Collins *et al.* 2014), and anti-Orb (Lantz et al. 1994). We used rabbit anti-Corolla at 1:3000 (gift from Scott Hawley), and mouse anti-Orb at 1:20 (DSHB clone 6H4 at 1:40 combined with clone 4H8 at 1:40). We used the following secondary antibodies: Goat anti-Mouse IgG2b Alexa Fluor 594 (Thermofisher # A-21145), Goat anti-Mouse IgG1 Alexa Fluor 488 (Thermofisher #A-21121), and Goat anti-Rabbit Alexa Fluor 647 (Thermofisher #A-21244).

Ovaries were imaged on a Leica Stellaris 5 confocal microscope using an HC PL APO 63x/1.4 NA Oil objective. Images were acquired using the Lighting module with an Airy pinhole size of 0.75 AU and the standard default settings dictated by the pinhole size. All images were deconvolved using the Leica Lightning internal software with default settings.

Measuring the two-oocyte phenotype

To determine how many oocytes were present in region 3, we used maximum projections of the z-stack. For Orb staining, we categorized a germarium as having one oocyte if Orb was concentrated in the cytoplasm of a single cell in region 3. We categorized it as having two oocytes if Orb was concentrated in the cytoplasm of two cells; in these cases, Orb was often found in the cytoplasm of two cells but centralized between the two nuclei. For Corolla staining, we categorized a germarium based on the presence of full-length Corolla in one or two nuclei in region 3; if there was fragmented Corolla staining in one nucleus, we did not categorize this as having two oocytes, consistent with previous work analyzing the two-oocyte phenotype (Joyce and McKim 2010) .

Statistical analyses

Fisher’s exact test with Bonferroni corrections for multiple tests were performed in GraphPad Prism version 10.2 for Mac (GraphPad Software, Boston, MA USA).
